# Third Molar Extraction: Irrigation and Cooling with Water or Sterile Physiological Solution: A Double-Blind Randomized Study

**DOI:** 10.3390/dj9040040

**Published:** 2021-04-01

**Authors:** Luca Sbricoli, Alessia Cerrato, Anna Chiara Frigo, Gastone Zanette, Christian Bacci

**Affiliations:** 1 Department of Neurosciences, Section of Clinical Dentistry, Unit of Oral Surgery and Pathology, University of Padova, 35122 Padova, Italy; luca.sbricoli@unipd.it (L.S.); cerrato.alessia92@gmail.com (A.C.); gastone.zanette@unipd.it (G.Z.); 2 Department of Cardiac, Thoracic, Vascular Sciences and Public Health, University of Padova, 35122 Padova, Italy; annachiara.frigo@unipd.it

**Keywords:** extraction, inflammation, irrigation, oral surgery, oral surgical procedures

## Abstract

Background: The present study aimed to ascertain whether any significant reduction in patients’ postoperative pain and inflammation could be achieved by using sterile physiological solution instead of normal water to irrigate the surgical field and cool the dental bur during third molar extractions. Methods: The study concerned 22 patients (11 females and 11 males) in good general health, who were referred to the Dental Clinic at Padova University hospital for lower third molar extractions. They were randomly assigned to two groups. Only the fluid used to irrigate the surgical field and cool the dental bur differed between the two study groups, being sterile physiological solution for group A, and mains water for group B. Postoperative pain, swelling, trismus and inflammation with high sensitivity CRP where measured and statistically evaluated. The numerosity of our sample was calculated on the grounds of an endpoint based on data in the literature. Results: Eighteen patients needed bilateral extractions, and 4 required only one extraction, so a total of 40 third molars were extracted. A sterile physiological solution was used to irrigate the surgical field in 20 extractions, while water was used in the other 20 cases. Data analysis with Wilcoxon test show no differences between the two groups (*p* < 0.05). Conclusions: no differences between groups for any of the parameters considered, after third molar extraction procedures undertaken using either sterile physiological solution or water for irrigation and cooling purposes.

## 1. Introduction

### 1.1. Impacted Third Molars: Definition, Epidemiology and Etiology

The surgical extraction of impacted third molars, especially of the lower jaw, is one of the most common clinical procedures in oral surgery [1,2,3,4,5,6,7,8,9,10]. Third molars may need to be extracted for various reasons, including: pericoronitis of partially impacted teeth; caries involving the third molar or the distal surface of the second molar; second molar root resorption; cystic neoformations; odontogenic tumors; orthodontic or prosthetic treatments; orthognathic surgical procedures; facial pain; and headache [11,12,13,14,15,16,17,18,19].

The generally-accepted age cut-off for third molar extractions is at 24 years old. This is because it has been demonstrated that the risk of permanent neurological impairment increases for patients beyond this age [2,11].

The complications of mandibular third molar extractions may involve: alveolitis or alveolar osteitis; infections; hematoma; hemorrhage; inferior alveolar nerve or lingual nerve damage; mandibular bone fractures; soft tissue lesions; iatrogenic tooth dislocations; complications affecting the temporomandibular joint (TMJ); and postoperative pain, trismus, and swelling [2,15,20,21,22,23,24]. Postoperative pain, orofacial tissue edema, and trismus are so common after third molar extractions that they are considered virtually routine [10]. They are due essentially to the tissue damage caused by the surgical procedure and the consequent inflammation.

The onset of postoperative pain coincides with the fading of the effect of local anesthesia, and usually peaks in intensity 12 h after the procedure [25]. The severity of this pain seems to correlate with the difficulty and duration of the surgical procedure, while no such correlation has been demonstrated for the severity of orofacial edema or trismus. On the other hand, a very strong association has been established between pain and trismus in this setting [25]. Trismus is usually caused by inflammation of the masticatory muscles [26,27] causing a spasm, often secondary to detachment of the mucoperiosteal flap. Other possible causes of trismus include TMJ trauma, direct medial pterygoid muscle trauma incurred during truncal block of the inferior alveolar nerve, and (more rarely) infections [26]. Orofacial tissue edema tends to peak at the end of the second postoperative day, and usually disappears by the fifth or sixth day after the procedure [7,25].

### 1.2. How Can We Reduce the Risk of Complications

Nowadays, approximately 90% of third molar extractions are completed without any major complications [9,28]. The reported incidence of major complications ranges from 4.6% to 30.9% [9,28]. Several risk factors have been identified, including: the patient’s age; a history of infections; the depth of dental impaction; the duration of the procedure; individual anatomical variability; smoking; the use of oral contraceptives; and the type of local anesthesia [2,9,28,29,30,31,32,33,34,35,36].

Since it has been demonstrated that the intensity and duration of postoperative pain increase with the difficulty and duration of the surgical procedure [25,37], expert surgeons and shorter procedures should reduce this type of complication to a minimum.

### 1.3. Aim of the Study

The present study aimed to ascertain whether any significant reduction in patients’ postoperative pain and inflammation could be achieved by using sterile physiological solution instead of normal water to irrigate the surgical field and cool the dental bur during third molar extractions.

The primary goal of the study was therefore to compare the severity of the pain experienced during the week after the extraction procedure, as rated by patients using a visual analog scale (VAS).

The goals of the study were amply explained to all participants and their informed consent was obtained.

## 2. Materials and Methods

### 2.1. Study Population

The study concerned 22 patients (11 females and 11 males) in good general health, who were referred to our Hospital Dental Clinic for lower third molar extractions between April 2016 and May 2017. Eighteen patients needed bilateral extractions, and 4 required only one extraction, so a total of 40 third molars were extracted. A sterile physiological solution was used to irrigate the surgical field in 20 extractions, while water was used in the other 20 cases.

The procedures were indicated for one or more of the reasons listed in the Introduction. Since some publications in the literature report different healing times and outcomes, and a higher risk of complications after the dental root has formed completely (at around 24 years old, see Introduction), this age threshold was taken into account. Half of the patients in our series were less than 24 years old, and the other half were older.

Patient inclusion criteria:

1. need for extraction of partially or totally impacted lower third molar(s);
2. tolerance of normal surgical procedures;
3. good general health;
4. informed consent to participation in the study.

Patient exclusion criteria:

1. uncontrolled periodontal disease;
2. uncontrolled diabetes;
3. bone diseases (Paget’s disease, therapy with bisphosphonates, multiple myeloma, metastatic cancer to bone);
4. a history of radiotherapy to the head and neck region;
5. need for systemic corticosteroids or other therapies that might interfere with postoperative recovery;
6. allergy to penicillin;
7. inability to return to follow-up visits and complete the study protocol as established by the investigators;
8. smoker patients were ineligible for this trial;
9. pregnancy and or breastfeeding;
10. acute inflammatory diseases.

Randomization:

Patients participating in the study were randomly assigned to group A (for surgical procedures during which physiological solution was used to irrigate the surgical field using) or group B (for procedures completed using mains water for irrigation purposes). The participants were randomly assigned by computer-generated table to either water or saline solution irrigation. To avoid unequal balance between the two groups a balanced random permuted block approach with 4-unit block size was used. One of the authors (A.C.) placed the fluids in specifically-prepared containers inside the dental unit, while the surgeon was blinded to the nature of the irrigation fluid being used. All third molar extractions were completed by the same surgeon (C.B.) to avoid any possibility of bias.

### 2.2. Surgical Procedure

Extractions were completed under intravenous conscious sedation [38]. The surgeon used the same type and quantity of local anesthetic, the same flap preparation (intrasulcular with disto-vestibular release starting from the distal surface of the first molar), and the same suture for all procedures. Only the fluid used to irrigate the surgical field and cool the dental bur differed between the two study groups, being sterile physiological solution for group A, and mains water for group B.

Patients were prescribed antibiotic therapy at home (amoxicillin plus clavulanic acid, in 1 g tablets consisting of 875 mg of amoxicillin +125 mg of clavulanate, twice a day for 6 days) and paracetamol (1000 mg) if necessary for pain control.

### 2.3. Postoperative Pain Rating

Participants were given a questionnaire to complete every day and return when they attended their follow-up visit one week after their third molar extraction.

They used a VAS to rate the severity of the pain they perceived after the surgical procedure on a horizontal scale 10 cm (100 mm) long, from “no pain” at one end to the “worst possible pain” at the other [3,39,40,41,42]. They were asked to rate their pain every day for seven days after the procedure. They were also asked to record any pain medication they took.

### 2.4. Postoperative Swelling

The following extraoral measurements were taken before the procedure (T1), 48 h afterwards (T2), and again one week after the extraction, before removing a patient’s stitches (T3).

- the distance from the tragus to the pogonion (the most prominent point on the chin);
- the distance from the tragus to the lateral canthus of the homolateral eye;
- the distance from the tragus to the labial commissure on the homolateral side;
- the distance from the gonion (the meeting point of lines tangent to the posterior margin of the ramus and to the inferior margin of the mandible) to the homolateral nasal wing;
- the maximal mouth opening range (distance between the incisal edge of the central upper and lower incisors) [3,42,43,44,45,46,47,48].

Measurements were taken using nylon 4/0 suture thread to adapt to the profile of the extraoral soft tissues. Then the length of suture thread was measured on a millimeter-scale ruler to obtain accurate distances.

### 2.5. Postoperative Trismus

The maximal mouth opening range was measured before the procedure (T1), and then 48 h (T2) and seven days (T3) afterwards.

### 2.6. Severity of Systemic Inflammation

Systemic inflammation was assessed by measuring patients’ serum C-reactive protein (CRP) levels, once immediately before the surgical procedure (T1), and then again 48 h afterwards (T2).

CRP is synthesized by the liver and by adipocytes, and is normally detectable in the bloodstream in very low concentrations, below 5–6 mg/L. But it can very rapidly reach very high levels (even hundreds of times higher than at the baseline) in the event of inflammatory processes and after certain surgical procedures. Immediately after third molar extractions, serum CRP levels are estimated to double every 8 h, then return to normal after approximately 7 days [49,50], differently from what can happen in dry alveolitis [51] or many other complications [52].

Participants’ serum CRP levels were assayed in 5 mL venous blood samples collected in appropriate test tubes and centrifuged at 3000× *g* revolutions per minute at the hospital within 2 h of sampling. In accordance with the guidelines of the American Heart Association, the test used at our Hospital is not sensitive to serum CRP variations in the range of 0–2.9 mg/L, while it identifies PCR concentrations in excess of 3 mg/L.

### 2.7. Statistical Analyses

The numerosity of our sample was calculated on the grounds of an endpoint based on data in the literature (BIBLIO). The expected difference between our study groups was 2 points on the VAS (on a scale from 0 to 10 points), with a standard deviation (SD) of 2, having established a power of 85% and a significance level of 5% using Mann-Whitney two-sided test (given the nature of the scale) [53]. The number of patients required for our study was distributed as follows: 20 patients in each of the two study groups (physiological solution versus water), half of them under 24 years old and the other half older. Patients’ demographic details and other basic characteristics are presented in the form of descriptive statistics.

The swelling measurements were described with median and range considering T1, T2 and the difference T1-T2. The T1-T2 difference was compared between the two groups with the Mann-Whitney test. The daily pattern of VAS was described with median and range. The analysis was performed considering the rank transformation and a mixed model ANOVA with group, time and group by time interaction and an undedermined covariance matrix.

A *p*-value < 0.05 was considered indicative of statistical significance. The statistical analysis was performed with SAS 9.4 (SAS Institute Inc., Cary, NC, USA) for Windows.

## 3. Results

The study was conducted on a sample of 40 extractions performed in patients from 18 to 37 years of age.

The third molar extraction procedure lasted from 18 to 46 min (mean 32 min) in group A (physiological solution), and from 20 to 45 min (mean 30 min) in group B (water).

### 3.1. Postoperative Pain

VAS ratings for postoperative pain by study group (Time—Water—Physiological solution—*p* value for group—*p* value for time—*p* value for group × time interaction) (see Table 1).

**Figure 1:**
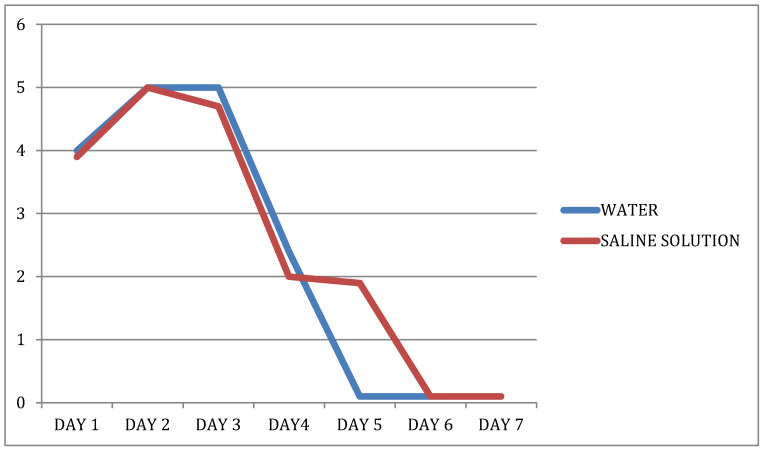
Vas values during time.

Trend of VAS ratings in the two groups over 7 days after the procedure (water/physiological solution—Postoperative days) (see Figure 1).

### 3.2. Postoperative Swelling and Trismus

Postoperative swelling measured in the two study groups (Water—Physiological solution—*p* value Wilcoxon’s test—Tragus to Pogonion—Tragus to lateral canthus—Tragus to labial commissure—Gonion to nasal wing—Mouth opening) (see Table 2).

### 3.3. Severity of Systemic Inflammation

Serum CRP levels, as a marker of systemic inflammation, did not change significantly during the postoperative period. The results are consequently reported using descriptive statistics.

There were only two patients whose serum CRP levels rose after third molar extraction, and in both cases they returned to within normal range within one week of the procedure

### 3.4. Local and Systemic Complications of the Procedure

There were no long-term complications of the third molar extraction procedure in our series. One patient reported fever (38.5 °C) on postoperative day 1. One patient experienced a lesion due to stretching of the labial commissure, which regressed within 5 days afterwards.

## 4. Discussion

Numerous publications in the literature describe efforts to reduce the complications of third molar extractions to a minimum.

Access flap design, osteotomy technique, the use of the retractors, postoperative pharmacological treatments, suture by first or second intention, the placement of a drainage tube, and leaving part of the tooth in situ are all aspects that have been considered. On the other hand, a literature review published in 2014 by the Cochrane Collaboration offered no suggestions concerning irrigation of the surgical field.

Third molar extractions involve sectioning of the skin or oral mucosa and thus placing the underlying tissues in direct communication with the surrounding oral environment, which is colonized by countless types of microorganism. Exposure to pathogens can raise the risk of infection, especially in the oral cavity.

In the present study, we proposed to see whether any significant reduction in patients’ postoperative pain and inflammation could be achieved by using sterile physiological solution instead of water for irrigating the surgical field and cooling the dental bur during the osteotomy and odontotomy involved in impacted third molar extractions. In other words, we wanted to test whether performing the procedure in a completely sterile environment benefited the patient—in terms of postoperative pain, swelling, trismus and inflammation—by comparison with a procedure completed in clean, but not sterile conditions.

Judging from our results, there were no statistically significant differences between the two study groups. The most plausible reason for this result probably lies in that the abundance of microbial populations in the oral cavity makes it impossible to obtain a genuinely and persistently sterile environment. It would therefore be pointless to use a sterile fluid instead of an appropriate, routinely-tested water supply.

There is also the matter of the disinfectant used to decontaminate the water piping in dental units, traces of which probably remain in minimal concentrations in the water used to irrigate the surgical field and cool dental drills. This might also be beneficial for the patient, though data to confirm such a hypothesis are lacking.

As shown in the graph (Figure 1), the level of pain that our two study groups perceived after their extraction procedure was almost always comparable. The exception concerns the VAS ratings obtained on the fifth postoperative day, when patients whose procedure had been completed using water for irrigation purposes (group B) rated their pain a mean 2 points lower than those treated using sterile physiological solution. Clinically, patients in group B seemed to be more comfortable by the fifth day, so statistical analyses were run on the VAS ratings in relation to time (Table 1). Here again, the differences obtained were not statistically significant.

The CRP levels measured in our sample showed no statistically significant variation, and are consequently presented here in the form of descriptive statistics alone (Table 3).

## 5. Conclusions

No statistically significant differences emerged between our two study groups for any of the parameters considered, after third molar extraction procedures undertaken using either sterile physiological solution or water for irrigation and cooling purposes.

## Figures and Tables

**Table 1:** Postoperative pain.

DAY	Water	Saline Solution	*p* Value Group	*p* Value Time	*p* Value Group × Time
1	4.00 (0.00–9.40)	3.85 (0.00–8.00)	0.5214	<0.0001	0.8355
2	5.00 (0.00–9.40)	5.00 (0.00–9.00)			
3	4.85 (0.00–8.00)	4.50 (0.00–8.50)			
4	3.00 (0.00–7.00)	2.15 (0.00–7.30)			
5	0.00 (0.00–5.00)	2.00 (0.00–7.00)			
6	0.00 (0.00–9.00)	0.00 (0.00–9.00)			
7	0.00 (0.00–7.00)	0.00 (0.00–8.00)			

**Table 2:** Postoperative swelling measured in the two study groups.

	Water			Saline Solution			*p* Value (Wilcoxon’s Test)
	T1	T2	Difference	T1	T2	Difference	
Tragus to Pogonion	14.60 (12.40–17.00)	15.35 (12.60–17.30)	−0.55 (−1.10–0.10)	14.05 (12.30–16.40)	14.75 (13.00–16.50)	−0.60 (−2.00–0.10)	0.5968
Tragus to lateral canthus	7.80 (7.00–8.60)	8.20 (7.0–9.30)	−0.50 (−0.90–0.00)	7.50 (6.80–8.50)	8.00 (7.00–9.00)	−0.40 (−1.10–0.00)	0.9133
Tragus to labial commissure	11.40 (9.80–12.60)	11.90 (10.40–13.80)	−0.40 (−1.80–0.00)	11.00 (9.80–12.00)	11.45 (10.30–12.40)	−0.40 (−0.70–0.00)	0.6224
Gonion to nasal wing	9.70 (7.20–11.00)	10.30 (8.70–11.50)	−0.40 (−2.00–0.20)	9.50 (7.60–10.50)	10.00 (8.50–11.40)	−0.35 (−1.90–0.00)	1.0000
Mouth opening	5.00 (4.00–6.50)	3.50 (2.00–6.00)	1.30 (0.40–3.50)	5.00 (4.00–6.00)	3.10 (2.00–4.20)	1.50 (0.30–3.40)	0.4640

**Table 3:** Variations in serum CRP levels.

20 patients	<2.9 mg/L (baseline)	<2.9 mg/L (postoperatively)
1 patient	<2.9 mg/L (baseline)	3.5 mg/L (postoperatively)
1 patient	<2.9 mg/L (baseline)	9.5 mg/L (postoperatively)
